# *Aspergillus fumigatus* Inhibits *Pseudomonas aeruginosa* in Co-culture: Implications of a Mutually Antagonistic Relationship on Virulence and Inflammation in the CF Airway

**DOI:** 10.3389/fmicb.2018.01205

**Published:** 2018-06-05

**Authors:** Emma Reece, Sean Doyle, Peter Greally, Julie Renwick, Siobhán McClean

**Affiliations:** ^1^Department of Clinical Microbiology, Trinity College Dublin – Trinity Centre for Health Science, Tallaght Hospital, Dublin, Ireland; ^2^Department of Biology, Maynooth University, Maynooth, Ireland; ^3^Department of Respiratory Medicine, The National Children’s Hospital, Tallaght Hospital, Dublin, Ireland; ^4^School of Biomolecular and Biomedical Science, University College Dublin, Dublin, Ireland

**Keywords:** co-colonization, cystic fibrosis, *Aspergillus fumigatus*, *Pseudomonas aeruginosa*, biofilm, inflammation, gliotoxin

## Abstract

Many cystic fibrosis (CF) airway infections are considered to be polymicrobial and microbe–microbe interactions may play an important role in disease pathology. *Pseudomonas aeruginosa* and *Aspergillus fumigatus* are the most prevalent bacterial and fungal pathogens isolated from the CF airway, respectively. We have previously shown that patients co-colonized with these pathogens had comparable outcomes to those chronically colonized with *P. aeruginosa*. Our objective was to examine the interactions between *A. fumigatus* and *P. aeruginosa*, specifically the effects of co-colonization on biofilm formation, virulence and host pro-inflammatory responses. Our findings suggest that co-infections of *A. fumigatus* and *P. aeruginosa* in the *Galleria mellonella* acute infection model showed that pre-exposure of larvae to sub-lethal inocula of *A. fumigatus* increased the mortality caused by subsequent *P. aeruginosa* infection. Co-infection of human bronchial epithelial cells (CFBE41o^-^) with both pathogens did not enhance IL-6 and IL-8 production beyond the levels observed following single infections. In addition, both pathogens stimulated cytokine secretion via the same two mitogen-activated protein kinases (MAPKs) signaling pathways, ERK and p38. Mixed species biofilms showed overall reduced biofilm development with crystal violet staining. Quantification by species-specific qPCR revealed that both pathogens had mutually antagonistic effects on each other. *A. fumigatus* supernatants showed strong anti-Pseudomonal activity and gliotoxin was the main active agent. Gliotoxin resulted in varying levels of anti-biofilm activity toward other bacteria commonly found in the CF airways. Gliotoxin produced by *A. fumigatus* colonizing the CF airways may have a significant impact on the CF airway microbiome composition with potential clinical implications.

## Introduction

Cystic fibrosis (CF) is the most common inherited life-shortening condition, characterized by mutations in the CF transmembrane conductance regulator (CFTR) gene. The resulting defective chloride secretion, altered airway surface liquid and impaired mucociliary secretion results in recurrent chronic respiratory infections (Haq et al., 2016). CF airway disease is characterized by a continuous cycle of persistent infection and inflammation contributing to morbidity and mortality in CF. Early detection and eradication of these infections remains a major challenge in CF lung disease management. *P. aeruginosa* is the leading cause of infection in patients with CF with approximately 28.4% being colonized by 71 months (Douglas et al., 2009). Prevalence in adults varies from 31 to 49.6% in recent reports (Salsgiver et al., 2016; Reece et al., 2017). Early intervention is paramount as *P. aeruginosa* is very difficult to eradicate once it has colonized the airways. *P. aeruginosa* is independently linked to worsened prognosis for CF patients; they have a decreased life expectancy of 30 years, compared with 40 years in non-colonized patients, experiencing a more rapid decline in pulmonary function with more frequent hospitalizations (Kosorok et al., 2001; Li et al., 2005). *P. aeruginosa* is a very accomplished bacterium and can adapt to life in the CF airway (Friman et al., 2013; Cullen and McClean, 2015).

Cystic fibrosis airway microbiology has recently been revolutionized by the discovery of complex communities of bacteria and fungi co-existing in the lungs of adults and children with CF (Sibley et al., 2011; Delhaes et al., 2012). These studies have highlighted a number of new and emerging microorganisms in CF airway disease (Bittar et al., 2008; Spicuzza et al., 2009) but have also verified the importance of common CF pathogens, such as *P. aeruginosa* (Cox et al., 2010; Klepac-Ceraj et al., 2010; Coburn et al., 2015) and *Aspergillus fumigatus* (Delhaes et al., 2012). Perhaps most importantly these metagenomic studies have confirmed that important CF pathogens do not colonize the airways in isolation. Exacerbation and disease progression in patients may be influenced by the interactions between these microorganisms in the CF airway. *P. aeruginosa* was identified in 54.1% of CF patients with persistent *A. fumigatus* infection and these co-colonized patients showed decreased lung function compared with patients clear of both pathogens (Amin et al., 2010). Direct contact between these organisms or indirect signaling may influence microbial pathogenicity (Duan et al., 2003).

Several studies have shown that *P. aeruginosa* interacts with other bacteria and fungi, inhibiting *Burkholderia cenocepacia* (Costello et al., 2014), *Candida albicans* (Brand et al., 2008) and *Scedosporium aurantiacum* (Kaur et al., 2015). *P. aeruginosa* increases its virulence when in co-culture with Gram-positive bacteria in *Drosophila* (Korgaonkar et al., 2013). *P. aeruginosa* pathogenicity was enhanced in a rat lung infection model by the presence of oropharyngeal flora and the increased virulence may be due to interspecies communication via autoinducer-2 (AI-2) mediated signaling (Duan et al., 2003). Both filamentation and biofilm formation of *A. fumigatus* was inhibited by *P. aeruginosa* through direct cell contact and by secreted molecules. Recently it was demonstrated that *P. aeruginosa* and *A. fumigatus* interact with each other via volatile communication mediators and that stimulation of *A. fumigatus* growth by *P. aeruginosa* did not require direct contact (Briard et al., 2016). *P. aeruginosa* culture filtrates inhibited and damaged *A. fumigatus* biofilms via metacaspase activation (Shirazi et al., 2016). *P. aeruginosa* elastase production is enhanced in the presence of *A. fumigatus* and may play a role in the damaging pathology associated with the CF lung and may explain why, at least in part, co-colonized patients have a poorer prognosis (Smith et al., 2015).

We have recently shown that 3.1% of Irish CF patients registered with the CF registry of Ireland were intermittently co-colonized with *A. fumigatus* and *P. aeruginosa* (Reece et al., 2017). In addition, co-colonization with both pathogens (even intermittently) resulted in comparable levels of hospitalizations, respiratory exacerbations and lung function reduction as in patients that were chronically colonized with *P. aeruginosa* alone which emphasizes the clinical significance of co-colonization with these microorganisms. The potential consequences of co-colonization with these two pathogens on infection and inflammation are poorly understood. We now report our investigations on the interactions between *A. fumigatus* and *P. aeruginosa* in terms of their virulence and the effect co-colonization has on the CF host immune system.

## Materials and Methods

### *P. aeruginosa* and *A. fumigatus* Strains and Growth Conditions

A number of clinical isolates and reference strains were used in this study and are listed in **Table 1**. All experiments were performed in a SafeFAST Class II Biosafety cabinet (Mason Technology, Dublin, Ireland), in compliance with BSL-II containment. Two *P. aeruginosa* isolates (PAco1 and PAco2) were isolated from the sputum of a CF patient co-colonized with *A. fumigatus* (AFco1 and AFco2). Sputum was homogenized by adding Sputasol 1:1 (Thermo Fisher Scientific) and incubated at 35°C for 15–20 min shaking. A volume of 50 μl was plated on CHROMAgar *Pseudomonas* (CHROMAgar^TM^) (LIP Diagnostics, Galway, Ireland) and incubated at 35°C for 48 h. Any blue/green colonies were picked and identity confirmed using Mass Spectrometry (Biomerieux Vitek). The AFref1, Δ*gli*G and complement Δ*gli*G^c^ strains were previously described (Davis et al., 2011). *A. fumigatus* strains were routinely cultured on Malt Extract agar (MEA) plates (LIP Diagnostics, Galway, Ireland) for 48 h at 37°C and conidia were harvested using phosphate buffer saline (PBS) (Sigma, Wicklow, Ireland) containing 0.1% Tween 80 (PBST) (Sigma, Wicklow, Ireland). Conidia were enumerated on a Fast Read 102 counting slide (Biosigma, Cona, Italy). Liquid cultures were grown in Malt extract broth and sterile filtered through a 0.22 μM Whatman filter (Sigma, Wicklow, Ireland) to harvest culture supernatants (CSNs). *A. fumigatus* CSNs were plated on MEA and cultured overnight to confirm the absence of conidia. All *P. aeruginosa* isolates were grown on Blood agar plates (LIP Diagnostics) and cultured in Luria-Bertani (LB) (Sigma, Wicklow, Ireland) broth at 37°C. Overnight cultures in LB broth were diluted to obtain an OD_600*nm*_ of 0.6 and sterile filtered through a 0.22 μM Whatman filter (Sigma, Wicklow, Ireland) to harvest CSNs. *P. aeruginosa* CSNs were plated on blood agar and cultured overnight to confirm absence of bacterial cells.

**Table 1:** Strains used in this study.

Strain name	Source	Strain type	Reference
PAref1	ATCC	Reference	ATCC 27853
PA27	CF patient	Mucoid	IST 27 De Soyza et al., 2013
PA27N	CF patient	Non-mucoid	IST 27N De Soyza et al., 2013
PAco1	CF patient	Co-colonizer	This study
PAco2	CF patient	Co-colonizer	This study
AFref1	ATCC	Reference	ATCC Af293
AFref2	ATCC	Reference	ATCC 26933
AF1	CF patient	Clinical	This study
AF2	CF patient	Clinical	This study
AFco1	CF patient	Co-colonizer	This study
AFco2	CF patient	Co-colonizer	This study
ΔgliG	ATCC	Mutant	Davis et al., 2011
ΔgliGc	ATCC	Complement	Davis et al., 2011
*Stenotrophomonas maltophilia*	ATCC	Reference	ATCC 17666
*S. aureus*	ATCC	Reference	ATCC 25923
*Haemophilus influenzae*	NCTC	Reference	8468
*A. baumannii*	Patient	Clinical	This study
*B. multivorans*	BCCM/LMG	Clinical	LMG 13010


### Virulence of Co-colonizing Pathogens in the *Galleria mellonella* Acute Infection Model

In order to establish bioburden to be injected, 10-fold serial dilutions of *A. fumigatus* strains were prepared in PBS and conidia counted on MEA plates. *P. aeruginosa* strains were cultured in LB broth for 24 h at 37°C, diluted to OD_600*nm*_ 0.6, pelleted and resuspended and 10-fold serial dilutions prepared of the neat sample (2 × 10^7^ CFU/ml) in 10 mM MgSO_4_ (Sigma, Wicklow, Ireland) and 50 μl aliquots spread on blood agar to confirm the bioburden. Prior to co-infection studies, kill curves (CFU/ml versus larval survival) for each *P. aeruginosa* or *A. fumigatus* strain were established by injecting serial dilutions of each *P. aeruginosa* or *A. fumigatus* strain into individual groups of larvae. The LD_50_ (CFU that resulted in 50% larval death after 24 h) of each strain was determined. For co-infection studies, groups of six healthy *G. mellonella* larvae (Livefoods Direct, Sheffield, United Kingdom) in the final instar larval stage weighing 0.25–0.35 g were selected and 10 μl of serial dilutions were injected into the hind left pro-leg using a 29-gauge needle (BD Biosciences, Oxford, United Kingdom) as described by Cullen et al. (2015). To establish the effect of *P. aeruginosa* infection in an *A. fumigatus* colonized model, *G. mellonella* were inoculated with non-lethal doses of *A. fumigatus* strains at 10^2^ conidia/10 μl (Supplementary Figure 2) 24 h prior to subsequent inoculation with *P. aeruginosa* strains at a concentration of 2 × 10^5^ CFU/10 μl. For experiments that required a second injection the hind right pro-leg was used. In all experiments two control groups were used, a group with no manipulation and a vehicle control group. Larvae were incubated at 37°C for the duration of each experiment and assessed every 24 h up to 7 days for viability as determined by their response to touch. The LD_50_ dose was determined from the percentage survival in three independent experiments.

### Culture of Bronchial Epithelial Cell Lines

CFBE41o^-^ bronchial epithelial cells (CFBEs) (Gruenert et al., 1988) were cultured in T-75 flasks coated with 1% (v/v) fibronectin, 1% (v/v) collagen, and 10% (v/v) bovine serum albumin with minimum essential media (MEM) supplemented with 10% fetal bovine serum (FBS), 1% (v/v) L-glutamine, 1% (v/v) non-essential amino acids and 1% (v/v) penicillin-streptomycin at 37°C in 5% CO_2_. Culture media was replenished every 2 days and the cells were sub-cultured by trypsin digestion when growth had reached 80–90% confluence. All culture reagents were supplied by Sigma (Wicklow, Ireland).

### Infection of CFBE Cells With *P. aeruginosa* and *A. fumigatus*

CFBE41o^-^ bronchial epithelial cells were seeded (4 × 10^5^ cells/ml) onto coated 24-well plates and incubated for 24 h at 37°C in 5% CO_2_ overnight. The media was changed to serum free media and the cells incubated for a further 24 h. Overnight cultures of *P. aeruginosa* were inoculated into 100 ml of LB broth and incubated at 37°C until OD_600*nm*_ 0.6 was reached. The CFU/ml was determined by plating on blood agar. The cultures were centrifuged at 4,500 × *g* for 10 min, resuspended in 1 ml MEM and applied to CFBE cells at 2 × 10^7^ CFU/ml [multiplicity of infection (MOI) of 50:1] in triplicate and incubated at 37°C with 5% CO_2_ (Costello et al., 2014). *A. fumigatus* cultures centrifuged at 4,500 × *g* for 10 min were resuspended in 1 ml MEM and conidia applied to CFBE cells at a concentration of 8 × 10^5^ conidia/ml (MOI of 2:1) in triplicate. The effect of *P. aeruginosa* and *A. fumigatus* infection on the viability of CFBE cells was assessed by the 3-(4,5-dimethylthiazol-2-yl)-2,5-diphenyltetrazolium bromide MTT Cell Proliferation Assay Kit (Cayman Chemical, Ann Arbor, MI, United States) according to the manufacturer’s instructions. The CFBE cells infected with *P. aeruginosa* PAref1 (MOI of 30:1, 50:1, 80:1, and 100:1) or *A. fumigatus* AFref1 (MOI of 1:1, 2:1, 5:1, 10:1) in triplicate for 24 h at 37°C in 5% CO_2._ Control cells were incubated with MEM only.

CFBE41o^-^ bronchial epithelial cells were co-infected with *P. aeruginosa* and *A. fumigatus* in the presence MAPK inhibitors as follows: 10 μM of SP600125 (JNK inhibitor), SB203580 (p38 inhibitor) or PD 98,059 (ERK inhibitor) (Sigma, Wicklow, Ireland) or combinations of these for 1 h at 37°C. The cells were subsequently infected with *P. aeruginosa* strains (MOI of 50:1) or *A. fumigatus* stains (MOI of 2:1) in triplicate. After 24 h infection the plates were centrifuged and the supernatant removed and stored at -80°C for the pro-inflammatory response assays. Experiments were carried out in triplicate on three independent occasions.

### Measurement of IL-6 and IL-8 in Cell Supernatants by Enzyme-Linked Immunosorbent Assay (ELISA)

IL-6 and IL-8 were measured by sandwich ELISA (BD Biosciences, Oxford, United Kingdom) according to manufacturer’s instructions. Briefly, the wells were coated with 100 μl/well of anti-human IL-6 or IL-8 monoclonal antibody and blocked for 1 h at room temperature with 200 μl/well assay diluent (PBS with 10% FBS). Standards (100 μl/well recombinant human IL-6 or IL-8) were prepared at a concentration range of 0–300 pg/ml for IL-6 and 0–200 pg/ml for IL-8 and plates were incubated for 2 h at room temperature. The detection antibody and Streptavidin-HRP reagent were added and incubated for a further 1 h at room temperature. The plates were washed with PBS with 0.05% Tween-20 (wash buffer) seven times, before addition of TMB substrate and incubation for 30 min in the dark. The stop solution (1M H_3_PO_4_) at 50 μl/well was added and the absorbance was read at 450 nm using Gen 5 plate reader (Bio-Tek). Experiments were carried out in triplicate on three independent occasions.

### Examination of Well Diffusion With *P. aeruginosa* and *A. fumigatus*

Well diffusion assays were performed on MEA plates with 4 × 10^5^ CFU/100 μl lawns of *P. aeruginosa* and 4 × 10^5^ CFU/100 μl *A. fumigatus* inoculated in the wells and vice versa. The zones of inhibition were examined after 48 h incubation at 37°C.

### Determination of Biofilm Formation Using the Crystal Violet Assay

*In vitro* biofilm formation was measured in round bottom plastic 96-well plates using crystal violet to measure the biomass using a modification of a previously described method (O’Toole and Kolter, 1998). Briefly, *P. aeruginosa* and *A. fumigatus* cells/conidia were seeded into a 96-well plate at 1 × 10^6^ CFU/ml per well. Plates were incubated at 37°C for 48 h. The effect of *A. fumigatus* conidia on *P. aeruginosa* biofilm formation was determined by adding *A. fumigatus* conidia to *P. aeruginosa* biofilms at time zero. The biomass of the mixed biofilm was then compared to the biomass of the sum of the *P. aeruginosa* and *A. fumigatus* untreated biofilm controls. *P. aeruginosa* cells were also seeded with 72 h CSNs from *A. fumigatus*. Sterile LB media was used in negative controls. Fresh media was added to the wells every 24 h to replenish nutrients. Unattached bacterial/fungal cells were removed by rinsing with 250 μl of distilled water five times. The wells were air dried for 45 min. The adherent cells were stained with 1% (w/v) Crystal Violet (Sigma, Wicklow, Ireland) at room temperature for 30 min and wells washed again. The dye was solubilized with 95% ethanol containing 0.05% (l/v) Triton X-100 (Sigma, Wicklow, Ireland). Biofilm formation was determined by reading the absorbance at 590 nm (Synergy H1 Multi-Mode Reader, Bio-Tek). Each experiment was carried out on three independent occasions, with six strain replicates per 96 well plate.

### Dual Quantification of Species-Specific Biofilm Growth by qPCR

*Pseudomonas aeruginosa* strains and *A. fumigatus* strains were each seeded at 1 × 10^6^ CFU/ml in a 6-well round bottom plate for 0, 24, 48, and 72 h. Co-cultured biofilms were created by seeding wells with 1 × 10^6^ CFU/ml of an *A. fumigatus* strain together with 1 × 10^6^ CFU/ml of a *P. aeruginosa* strain. *P. aeruginosa* biofilms were also seeded with 24, 48, and 72 h *A. fumigatus* CSNs. Wells containing sterile LB media were used as negative controls. The plates were incubated at 37°C and spent LB broth replenished every 24 h. At 24, 48, and 72 h the broth was aspirated and the biofilms removed by scraping and transferred to a fresh tube. All biofilm experiments were performed in triplicate on three independent occasions. The biofilms were vortexed and 50 μM propidium monoazide (PMA) (Biotium, Fremont, CA, United States) was added prior to incubation in the dark for 30 min. The samples were irradiated with light at 464–476 nm using the phAST Blue activation system (GenUIL, Barcelona, Spain) for 15 min to activate the PMA and exclude DNA from non-viable cells (Nocker et al., 2006). The samples were then centrifuged at 5,000 × *g* for 10 min and pellets resuspended in 500 μl PBS before centrifuging again at 5,000 × *g* for 10 min.

Pelleted cells were resuspended in 200 μl molecular grade water (Sigma, Wicklow, Ireland) before transferring to sterile screw-capped tube containing 0.3 g sterile acid washed glass beads (Sigma, Wicklow, Ireland) and vortexing in a horizontal multi-vortexer for 180 s at 2500 rpm. The tubes were centrifuged at 420 × *g* for 1 min, supernatants transferred to fresh centrifuge tube and the DNA eluted using the High Pure PCR Template Preparation kit (Roche, Clare, Ireland) according to the manufacturer’s instructions. A negative extraction control was performed for each batch of DNA extractions where the entire DNA extraction protocol was performed on 200 μl molecular grade water.

*Pseudomonas aeruginosa* was specifically quantified by multiplex qPCR with primers for the *ecfX* and *gyrB* genes and hydrolysis probes *ecfX*-TM and *gyrB*-TM (Qin et al., 2003; Anuj et al., 2009). *A. fumigatus* was quantified using primers and probed for the internal transcribed spacer 1 (ITS1) region (Walsh et al., 2011) (Supplementary Table 1) All primers and probes were supplied by Eurofins Genomics, Ebersberg, Germany. The master mix was composed of 10 μl of TaqMan Gene Expression (Biosciences, Dublin, Ireland), 0.4 μM of each primer, 0.16 μM of each hydrolysis probe, and 4 μl of DNA in a final reaction volume of 20 μl. Cycling was performed on QuantStudio^TM^ 5 Real Time PCR System (Applied Biosystems, Dublin, Ireland), with an initial hold at 95°C for 15 min, followed by 45 cycles at 95°C for 15 s, and 60°C for 1 min, with a C_T_ of 35 being the threshold. A negative control consisting of master mix without DNA was included in each qPCR run alongside the negative extraction control. DNA extracted from 10-fold serial dilutions (10^1^–10^6^ CFU/ml) of either AFref1 or PAref1 was used to generate a standard curve and to act as a positive control. The standard curves of different reference concentrations were plotted against the corresponding C_T_ values and used to determine the concentrations of unknown samples. A *ctrA* plasmid was amplified in duplicate on each qPCR run (Supplementary Table 1) as a reaction control. Each sample was run in duplicate and samples were considered as positive if both duplicates showed C_T_ values below 35. The amplification efficiency of the qPCR was calculated from the slope of the standard curve using the following equation; *E* = 10 ^(-1/slope).^

### Bacterial Biofilms Treated With Gliotoxin

*Pseudomonas aeruginosa*, *Staphylococcus aureus*, *Stenotrophomonas maltophilia*, *B. cenocepacia*, *Acinetobacter baumannii*, *H. influenzae*, and *Escherichia coli* were seeded at 1 × 10^6^ CFU/ml per well in a 96 well plate and a range of concentrations of gliotoxin (Gt) (0.2–1 μM) were added to each well and incubated at 37°C for 48 h. HPLC analysis showed the concentration of gliotoxin produced by *A. fumigatus* at 72 h equates to approximately 0.8 μM commercial Gt (Coughlan et al., 2012), which was therefore used in this study. Fresh media was added to the wells every 24 h to replenish nutrients. Crystal violet assays were carried out to determine if Gt reduced the biomass of the biofilms of each of these pathogens.

### Data Analysis

Two-way ANOVAs with Bonferroni multiple comparisons tests were used to analyze the cytokine and MAPK assays. One-way ANOVAs with Dunnett’s multiple comparisons tests were used to analyze the crystal violet, the MTT and qPCR assays. LD_50_ values were compared via Student *t*-tests to determine statistical significance. *P*-values < 0.05 were considered significant. All results were analyzed using GraphPad Prism 6 and 7. The qPCR data was analyzed using the Quantstudio Design and Analysis software (Applied Biosystems).

## Results

### Pre-exposure of *G. mellonella* to a Non-lethal Dose of *A. fumigatus* Significantly Increased Larvae Mortality due to *P. aeruginosa* in a Strain-Dependent Manner

As an initial visual evaluation of the potential for the two pathogens to interact with each other, we evaluated inhibition of growth in well diffusion assays. PA27 and PA27N inhibited *A. fumigatus* strains with zone of inhibition (ZOI) apparent around the wells (Supplementary Figures 1A–D). The two clinical *P. aeruginosa* strains (PAco1 and PAco2) which were isolated from patients co-colonized with *A. fumigatus* also caused ZOIs in the growth of their respective co-colonizing strains AFco1 and AFco2 (Supplementary Figures 1E,F).

In order to examine whether these two pathogens alter their virulence when co-infecting with the other, their virulence in the *G. mellonella* acute infection model was evaluated. A non-lethal dose for *A. fumigatus* was first determined. All *A. fumigatus* strains caused larval mortality in a dose dependent fashion (Supplementary Figure 2) and day 4 was identified as the optimal time-point for distinguishing subtle differences between strains. There was 100% survival of the *G. mellonella* after 3 days when injected with 10^2^ conidia/ml for all *A. fumigatus* strains. Therefore, 10^2^ conidia/ml of *A. fumigatus* were considered a non-lethal dose after 3 days in future experiments.

**FIGURE 1:**
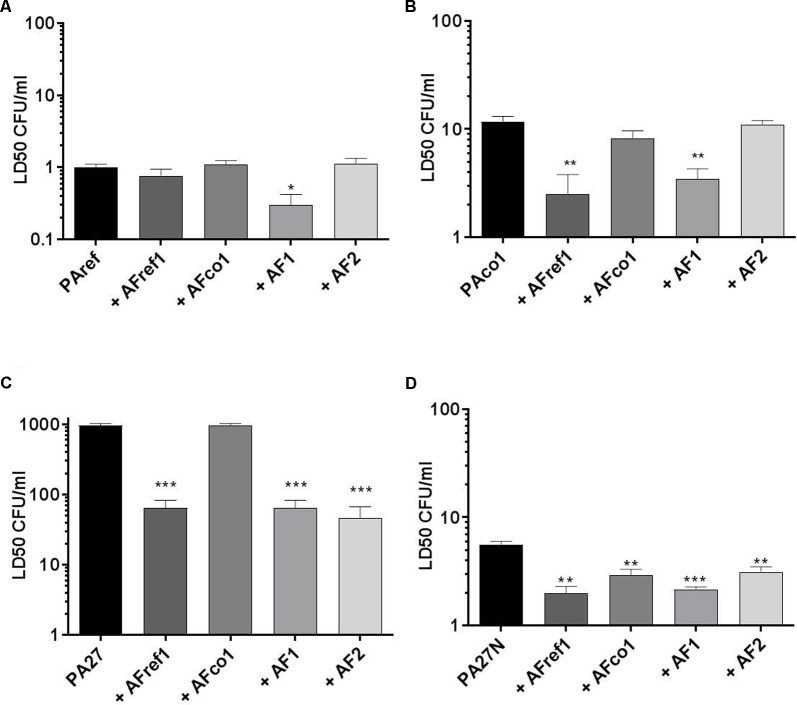
Virulence of *P. aeruginosa* and *A. fumigatus* co-infections in the *G. mellonella* infection model. LD_50_ of *P. aeruginosa* **(A)** PAref, **(B)** PAco1, **(C)** PA27, and **(D)** PA27N following pre-exposure to non-lethal doses of *A. fumigatus* strains in the *G. mellonella* infection model. Experiments were carried out on three independent occasions and error bars represent standard error. ^∗^*p* ≤ 0.05, ^∗∗^*p* ≤ 0.01, ^∗∗∗^*p* ≤ 0.001.

*P. aeruginosa* reference strain PAref, showed an LD_50_ of 1 CFU/ml. Pre-exposure of larvae to a non-lethal dose of *A. fumigatus* strain AF1 significantly reduced the LD_50_ of *P. aeruginosa* PAref (*p* = 0.012) compared to PAref alone indicating an apparent increase in virulence of PAref (**Figure 1A**). Consistent with this, the LD_50_s of the PAco1 strain was also reduced in the presence of AFref (*p* = 0.007) and AF1 (*p* = 0.003) (**Figure 1B**). The mucoid *P. aeruginosa* strain, PA27, showed a much higher LD_50_ than its non-mucoid conversion isolate and pre-exposure of larvae to all but one *A. fumigatus* strain significantly reduced LD_50_s of *P. aeruginosa* (*p* = 0.0002 for all three comparisons) compared to PA27 alone (**Figure 1C**). The LD_50_s of the non-mucoid *P. aeruginosa* strain, PA27N, reduced in the presence of AFref (*p* = 0.0018), AFco1 (*p* = 0.0082), AF1 (*p* = 0.001) and AF2 (*p* = 0.008) (**Figure 1D**). Interestingly pre-exposure to the co-colonizing strain, AFco1 did not reduce the LD_50_ of PA27, PAref or PAco1 (**Figures 1A–C**). Overall pre-exposure of larvae to a non-lethal dose of the majority of *A. fumigatus* isolates resulted in an apparent increase in virulence of the individual *P. aeruginosa* isolates when co-infecting in *G. mellonella* and this increase seems to be strain specific.

### *P. aeruginosa* and *A. fumigatus* Co-infection Altered the Pro-inflammatory Response in CF Epithelial Cells

**FIGURE 2:**
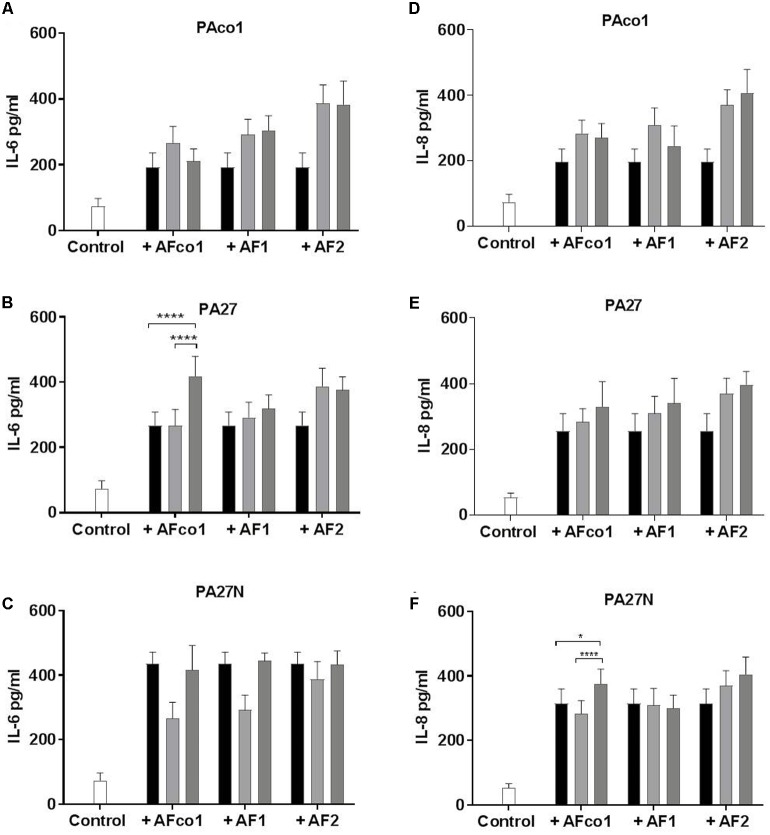
IL-6 and IL-8 secretion from CFBE cells in response to co-infection of *P. aeruginosa* cells and *A. fumigatus* conidia. Secretion of IL-6 pg/ml **(A–C)** and IL-8 pg/ml **(D–F)** from CFBE cells in response to co-infections of *P. aeruginosa* cells PAco1 **(A,D)**, PA27 **(B,E)**, PA27N **(C,F)** and *A. fumigatus* conidia. Black bars represent *P. aeruginosa* alone, light gray bars *A. fumigatus* alone and dark gray bars co-exposure of the CFBEs. Experiments were carried out on three independent occasions in triplicate and error bars represent standard deviation. ^∗^*p* ≤ 0.05, ^∗∗∗∗^*p* ≤ 0.0001.

Given that both *P. aeruginosa* and *A. fumigatus* elicit a profound pro-inflammatory response at the lung epithelium, we wanted to examine the effects of co-colonization on the inflammatory response of CFBE cells relative to *A. fumigatus* or *P. aeruginosa* isolates alone. Only two combinations resulted in significantly increased IL-6 and IL-8 secretion (**Figure 2**). There was a significant increase in IL-6 production from CFBE cells when infected with co-cultures of PA27 and AFco1 compared to PA27 or AFco1 alone (*p* ≤ 0.0001 and *p* ≤ 0.0001, respectively) (**Figure 2B**). An increase in IL-8 release was also observed when CFBE cells were co-infected with PA27N and AFco1 compared to PA27N (*p* = 0.013) or AFco1 alone (*p* < 0.0001) (**Figure 2F**). In contrast, IL-6 and IL-8 production from CFBE cells when infected with all other co-cultures showed the cytokine released did not increase compared to either *P. aeruginosa* or *A. fumigatus* alone (**Figures 2A,C–E**). The lack of enhancement of pro-inflammatory responses for the majority of tested co-infections suggests that co-infections of *A. fumigatus* and *P. aeruginosa* may not generally further exacerbate the inflammatory response. Uninfected controls produced on average of 74 pg/ml IL-6 and 53 pg/ml IL-8. The CFU of *P. aeruginosa* and *A. fumigatus* used to infect CFBE cells (MOI 50:1 and 2:1, respectively) were not cytotoxic to the CFBE cells (Supplementary Figure 3) ensuring that the lack of enhancement of cytokine release was not due to a loss in viability of the CFBE cells.

### *P. aeruginosa* and *A. fumigatus* Signaled via the Same MAPK Pathway

In order to further investigate why the majority of co-infections did not have an additive effect on the secretion of IL-6 and IL-8, we examined the signaling mechanisms of both pathogens. All four co-cultures elicited reduced IL-6 secretion in the presence of either ERK or p38 inhibitors and these levels were further reduced to that of the uninhibited controls when ERK and p38 inhibitors were used in combination (*p* ≤ 0.0001 in all four co-cultures). These reductions in IL-6 secretion varied from 16 to 26% relative to the corresponding uninhibited samples, suggesting the co-culture of both *P. aeruginosa* and *A. fumigatus* induce an IL-6 response via the ERK and p38 MAPK pathways (Supplementary Figures 4A–D).

In all co-culture combinations there was also a significant decrease in IL-8 secretion in the presence of the combination of ERK and p38 inhibitors. The IL-8 secretion was reduced to 11–19% (*p* ≤ 0.0001) of the uninhibited samples which suggests that the IL-8 secretion elicited by the co-cultures of *P. aeruginosa* and *A. fumigatus* also signals via the ERK and p38 pathways (Supplementary Figures 4E–H). When the CFBEs were infected with the co-cultures PA27 and AF1 in the presence of JNK inhibitors there was only a slight decrease in IL-8 production to 89% (*p* = 0.04) (Supplementary Figure 4E). Overall the impact of the JNK pathway was minimal.

### *A. fumigatus* and *P. aeruginosa* Grown in Co-cultured Biofilms Displayed Mutually Antagonistic Behavior

Initial well diffusion assays suggested that there was a degree of antagonism between the two pathogens, but this was not quantifiable. In order to quantitate the overall level of antagonism mixed biofilms of *A. fumigatus* conidia and *P. aeruginosa* cells were prepared. It was apparent the resulting biofilm biomass (as measured by crystal violet) was significantly reduced to 60–79% relative to the sum of single species biofilm controls for all isolates tested (range *p* = 0.0001–0.011) (**Table 2**). These results suggest that when *P. aeruginosa* and *A. fumigatus* were cultured together in a co-cultured biofilm, biofilm formation was significantly impaired relative to that formed by individual species. Overall mixed biofilms between any of the *P. aeruginosa* strains and any of the *A. fumigatus* strains showed reduced total biofilm formation, compared with the individual biofilm on its own.

**Table 2:** Reduction in mixed species biofilm formation in comparison to single species biofilms as measured by the crystal violet assay.

	Inhibition in biofilm formation (%) ± standard deviation^∗^				
	
	PA27	PA27N	PAco1	PAco2	PAref
AFref1	71.8 ± 2.6	68.4 ± 3.4	66.3 ± 0.6	64.2 ± 4.2	60.5 ±6
AF1	77.4 ± 4	74.2 ± 3.3	73.5 ± 1.5	72.8 ± 1.3	66.4 ± 3.6
AF2	79.1 ± 3.3	69.4 ± 1.3	74.6 ± 1.1	68.2 ± 4.4	62.5 ± 6
AFco1	74.2 ± 7.7	68 ± 4.1	62.3 ± 5.3	70.2 ± 2.7	67.1 ± 5.2
AFco2	77.8 ± 1.3	68.6 ± 2.4	66.2 ± 4.4	63.3 ± 2.2	62.9 ± 4.4


*^∗^*P* ≤ 0.03. The % inhibition was determined from the difference between the OD value of each mixed biofilm and the sum of the OD values of biofilm formed by the corresponding individual species.*

In order to quantify the inhibitory effects at the species level, species-specific qPCR was carried out to determine the reduction of *A. fumigatus* biofilms by *P. aeruginosa* and vice versa in the mixed biofilms. Optimization of qPCR resulted in amplification efficiencies of *P. aeruginosa* and *A. fumigatus* of 105.3% (+4.97% and ranged between 90.6 and 112.2% efficiency). The *R*^2^ values of the standard curves varied from 0.9807 to 0.9985 and all duplicates were within 0.5 Ct values of each other, indicating high consistency across replicates.

**FIGURE 3:**
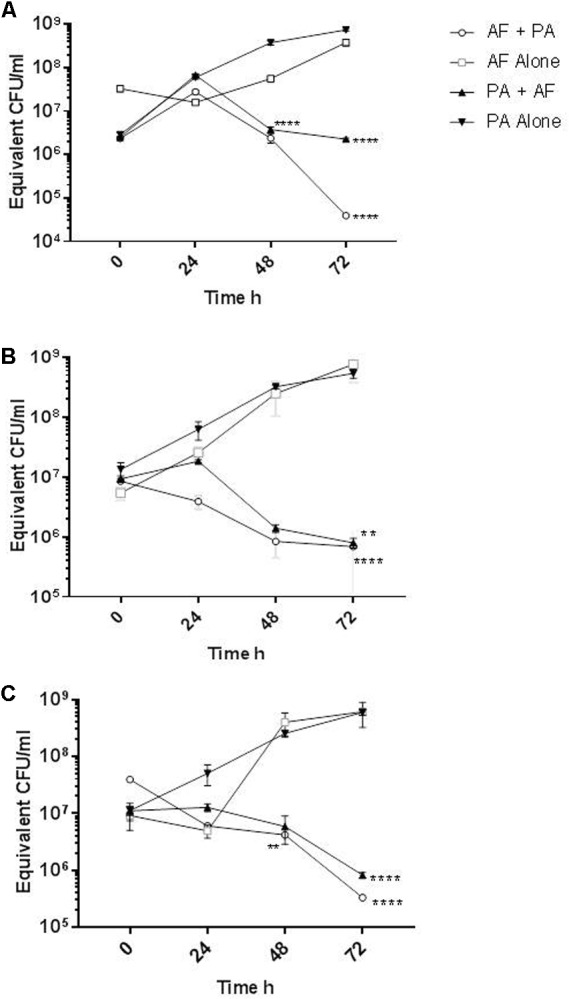
Reduction of *A. fumigatus* and *P. aeruginosa* biofilm when in mixed species biofilms. Quantification of biofilm biomass as measured by qPCR of *A. fumigatus* biofilm alone (open squares) and when in a co-cultured biofilm with *P. aeruginosa* (open circles) or *P. aeruginosa* biofilm alone (inverted triangles) and when in a co-cultured biofilm with *A. fumigatus* (upright triangles). **(A)** AFref1 and PAref individually and in co-cultured biofilm; **(B)** AFco1 and PAco1 individually and in co-cultured biofilm; **(C)** AFco2 and PAco2 individually and in co-cultured biofilm. Experiments were carried out on three independent occasions and error bars represent standard error. ^∗∗^*p* ≤ 0.01, ^∗∗∗∗^*p* ≤ 0.0001.

*Aspergillus fumigatus*-specific qPCR confirmed that *A. fumigatus* AFref1 biofilm increased over time from seeding at 0 to 72 h culminating in an estimated 3.8 × 10^8^ equivalent conidia/ml. In the mixed biofilm PAref did not cause any reduction in AFref1 biomass up to 48 h, however, at 72 h *A. fumigatus* was significantly reduced in the co-cultured biofilm from 3.8 × 10^8^ equivalent conidia/ml to 3.9 × 10^4^ CFU/ml (*p* ≤ 0.0001) (**Figure 3A**), demonstrating that the presence of *P. aeruginosa* in a co-cultured biofilm with *A. fumigatus* inhibited the viable fungal biofilm.

The biofilm formed by *P. aeruginosa* reference strain PAref in isolation increased over 72 h culminating in 7.5 × 10^8^ equivalent CFU/ml. *P. aeruginosa* biofilm formation was significantly reduced when in a mixed biofilm with AFref1 at 48 h by over 2 log (*p* ≤ 0.0001) and at 72 h (*p* ≤ 0.0001) (**Figure 3A**), demonstrating that the presence of *A. fumigatus* in a co-cultured biofilm with *P. aeruginosa* inhibited the viable bacterial biofilm.

Similar results were observed with mixed biofilms of the co-colonizing clinical strains. *A. fumigatus* AFco1 biofilm formation increased over 72 h culminating in an estimated 7.6 × 10^8^ equivalent conidia/ml. At 72 h *A. fumigatus* was significantly lower in the co-cultured biofilm at 6.8 × 10^5^ equivalent conidia/ml (*p* ≤ 0.0001) (**Figure 3B**). The biofilm of PAco1 increased over time culminating in 5.4 × 10^8^ equivalent CFU/ml at 72 h. *P. aeruginosa* biofilm formation was significantly less when in a mixed biofilm with AFref1 at 72 h at 8 × 10^5^ equivalent CFU/ml (*p* = 0.0024) (**Figure 3B**). *A. fumigatus* AFco2 biofilm increased over 72 h culminating in an estimated 6.1 × 10^8^ equivalent conidia/ml. In the mixed biofilm AFco2 was significantly reduced when in a mixed biofilm with PAco2 at 48 h and at 72 h (*p* = 0.0085, **Figure 3C**). The biofilm of PAco1 increased over time culminating in 5.9 × 10^8^ equivalent CFU/ml at 72 h. *P. aeruginosa* biofilm formation was significantly reduced when in a mixed biofilm with AFref1 at 72 h to 8.2 × 10^5^ equivalent CFU/ml (*p* ≤ 0.0001) (**Figure 3C**). It is apparent that there is a strong mutual antagonistic relationship present between *A. fumigatus* and *P. aeruginosa* in a mixed biofilm.

### *A. fumigatus* CSNs Inhibited *P. aeruginosa* Biofilms

*Pseudomonas aeruginosa* biofilms were treated with 72 h CSNs from *A. fumigatus* and the biomass measured by crystal violet. The bacterial biomass was reduced to 72–88% of the untreated biofilm controls (**Table 3**), indicating that *A. fumigatus* CSN can significantly decrease bacterial biofilm growth.

**Table 3:** Reduction of *P. aeruginosa* biofilm formation when treated with 72 h CSNs from *A. fumigatus.*

	Inhibition in biofilm formation (%) ± standard deviation^∗^				
	
	AFref1	AF1	AF2	AFco1	AFco2
PA27	77.2^#^ ± 12.5	77.6 ± 9.8	72.8^#^ ± 6.1	84.1^#^ ± 22.7	74.2 ± 13.8
PA27N	80.0 ± 4.8	83.6 ± 8.4	76.8 ± 2.5	79.3 ± 4.2	74.9 ± 4.8
PAco1	81.0^#^ ± 1.4	73.6 ± 9.6	78.7 ± 4.6	75.2 ± 3	80.0 ± 12.8
PAco2	85.4^#^ ± 14.1	83.2^#^ ± 12.2	86.4^#^ ± 12	85.8^#^ ± 26	88.3^#^ ± 22.9
PAref1	81.8 ± 3.2	77.6 ± 2.5	75.8 ± 2.4	77.1 ± 1.7	76.5 ± 10.4


*^∗^*P* ≤ 0.03, ^#^not significant. Results are presented as the % that the biofilms were reduced to in the presence of CSN relative to control untreated biofilms.*

**FIGURE 4:**
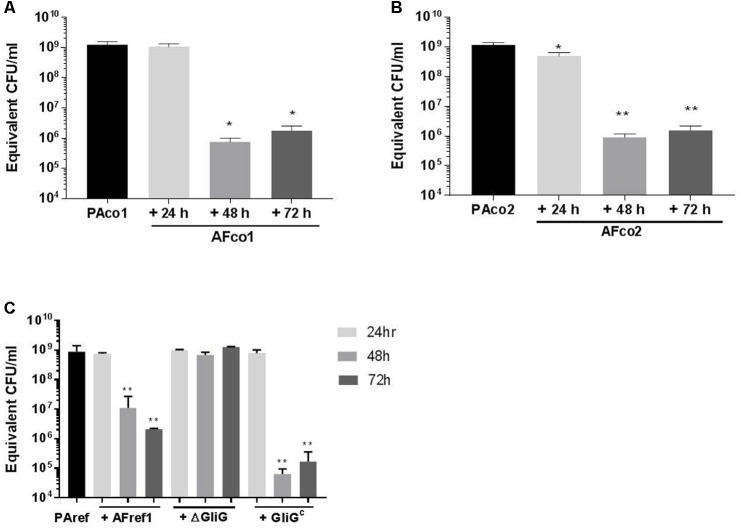
Reduction of *P. aeruginosa* biofilm when exposed to *A. fumigatus* CSN. Quantification of 48 h *P. aeruginosa* **(A)** PAco1, **(B)** PAco2, **(C)** PAref1 biofilms when exposed to **(A)** AFco1 **(B)** AFco2 **(C)** AFref1, AFref1 Δ*Gli*G and AFref1 Δ*Gli*G^c^ 24, 48, and 72-h-old CSNs as determined by qPCR. Experiments were carried out on three independent occasions and error bars represent standard deviation. ^∗^*p* ≤ 0.05, ^∗∗^*p* ≤ 0.01, ^∗∗∗^*p* ≤ 0.001, ^∗∗∗∗^*p* ≤ 0.0001.

In order to quantify the reduction of *P. aeruginosa* biofilm formation by *A. fumigatus* CSN, qPCR was utilized and the bacterial cells were treated with 24, 48, and 72-h-old *A. fumigatus* CSN and grown for 48 h. When PAco1 biofilms (1.01 × 10^9^ CFU/ml) were treated with 24-h-old AFco1 CSN there was no biofilm decrease, however, when the bacterial biofilm was exposed to 48 h and 72 h AFco1 CSN the viable *P. aeruginosa* biofilm decreased to 8.6 × 10^5^ CFU/ml (*p* = 0.0183), and 1.9 × 10^6^ CFU/ml (*p* = 0.0184), respectively. **Figure 4A**, suggesting 48-h and 72-h-old AFco1 CSNs inhibited the growth of *P. aeruginosa* viable biofilms.

When PAco2 biofilms were treated with 24-h-old AFco2 CSN the biofilm decreased from 1.1 × 10^9^ CFU/ml to 8.5 × 10^8^ CFU/ml (*p* = 0.028) and when the bacterial biofilm was exposed to 48 h and 72 h AFco2 CSN the viable biofilm decreased to 6.7 × 10^5^ CFU/ml (*p* = 0.001) and 2.2 × 10^6^ CFU/ml (*p* = 0.001), respectively (**Figure 4B**), suggesting CSN from *A. fumigatus* inhibited growth of *P. aeruginosa* viable biofilms. PAref biofilms decreased from 7.55 × 10^8^ CFU/ml to 1.1 × 10^7^ CFU/ml and 2.1 × 10^6^ CFU/ml (*p* = 0.0012 and *p* = 0.0011, respectively) when treated with 48 h and 72 h AFref1 CSNs (**Figure 4C**) suggesting 48-h and 72-h-old AFref1 CSNs inhibited growth *P. aeruginosa* viable biofilms.

### Gliotoxin Produced by *A. fumigatus* Inhibited *P. aeruginosa* Biofilm Formation

Gt is known to be produced by *A. fumigatus* in high quantities in 48 h and 72 h CSNs but in relatively low concentration at 24 h (Reeves et al., 2004) leading us to investigate whether Gt was causing this anti-Pseudomonal and anti-biofilm effect. *P. aeruginosa* cells were treated with CSNs from the Δ*gli*G mutant AFref1 strain and viable *P. aeruginosa biofilms* were quantified by qPCR after 48 h. The CSNs from the Δ*gli*G mutant strain did not inhibit *P. aeruginosa* biofilm formation suggesting Gt is the main inhibitory agent secreted at 48 h and 72 h by *A. fumigatus* and has anti-pseudomonal and anti-biofilm effects. Exposure to CSN from the complemented strain AFref1Δ*gli*G^c^ strain restored inhibition (**Figure 4C**).

### Gliotoxin Inhibited Bacterial Biomass

**FIGURE 5:**
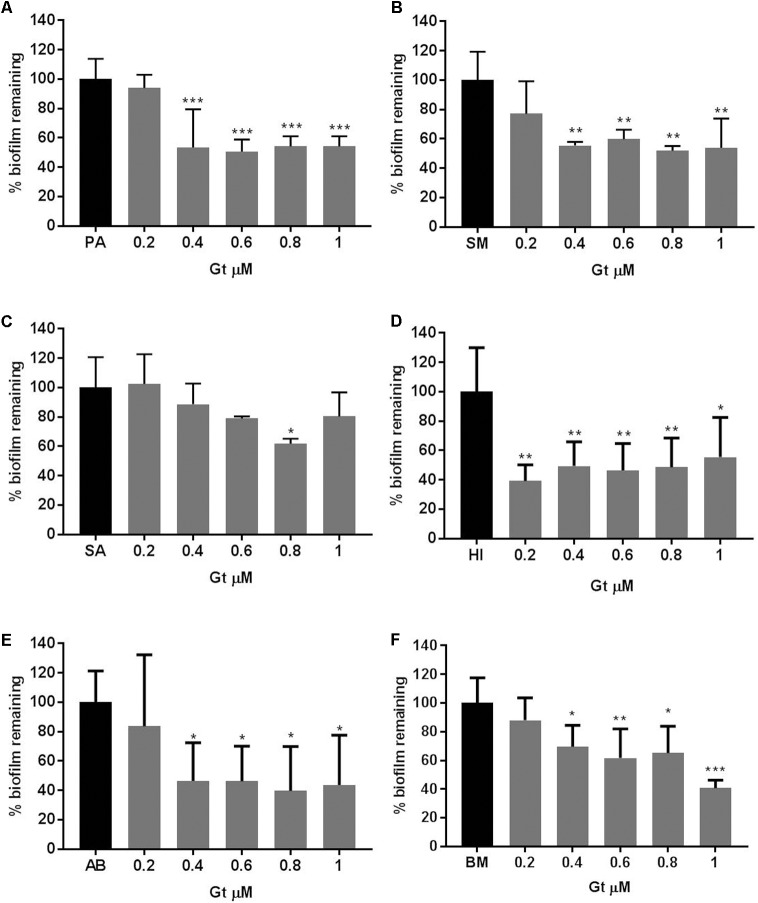
Reduction of bacterial biofilm formation when treated with gliotoxin. Percentage reduction of **(A)** PA: *P. aeruginosa*, **(B)** SM: *S. maltophilia*, **(C)** SA: *S. aureus*, **(D)** HI: *H. influenzae*, **(E)** AB: *A. baumannii*, **(F)** BM: *B. multivorans* biofilms when treated with gliotoxin (Gt) at concentrations of 0.2–1 μM measured by crystal violet assay. Experiments were carried out on three independent occasions and error bars represent standard deviation. ^∗^*p* ≤ 0.05, ^∗∗^*p* ≤ 0.01, ^∗∗∗^*p* ≤ 0.001, ^∗∗∗∗^*p* ≤ 0.0001.

In order to examine whether the gliotoxin inhibitory action was specific to *P. aeruginosa* or it had general anti-biofilm effect, a number of bacterial biofilms that are commonly found in the CF airways were treated with Gt at concentrations of 0.2–1 μM for 48 h. *P. aeruginosa* biofilms decreased significantly when treated with Gt at concentrations of 1–0.4 μM (*p* = 0.0001–0.0002) (**Figure 5A**). The biomass of *S. maltophilia* decreased significantly when treated with Gt at concentrations of 1–0.4 μM (*p* = 0.0012–0.0046) (**Figure 5B**). Gt also significantly decreased the biofilms of *S. aureus* (*p* = 0.024), *H. influenzae* (*p* = 0.0015–0.0146), *A. baumannii* (*p* = 0.0218–0.0426) and *B. multivorans* (*p* = 0.0003–0.0403) (**Figures 5C–F**). These results indicate gliotoxin has broad-spectrum antibacterial activity.

## Discussion

The airways of patients with CF lung diseases host a diverse and complex community of microorganisms (Beck et al., 2012; Renwick et al., 2014). Inter-, intra-species and cross-kingdom competition and/or cooperation within the airway microbiome is likely to influence disease progression (Sibley et al., 2009; Drescher et al., 2014). *P. aeruginosa* and *A. fumigatus* are isolated together from the sputum of CF patients and it has been well established that *P. aeruginosa* inhibits *A. fumigatus in vitro* (Kerr et al., 1999; Mowat et al., 2010; Ferreira et al., 2015; Shirazi et al., 2016; Anand et al., 2017) In contrast, much less is known about the impact of *A. fumigatus* on *P. aeruginosa*. We now report that *P. aeruginosa* and *A. fumigatus* have a mutually antagonistic relationship both in planktonic and biofilm growth forms and provide evidence that this may contribute to increased mortality in the *G. mellonella* infection model and reduced inflammation in CFBE epithelial cells. In particular, we report for the first time the anti-pseudomonal and anti-biofilm effects of *A. fumigatus* on *P. aeruginosa*.

We studied a combination of eleven clinical isolates and reference strains, including co-colonizing clinical strains AFco1, AFco2 with their respective co-colonizers, PAco1 and PAco2 as it is widely accepted that different strains of the same species can be phenotypically quite different. For instance, there is a broad-strain dependent variation of the immune response elicited to *A. fumigatus* infection indicating that different isolates have diverse virulence (Rizzetto et al., 2013). *P. aeruginosa* mucoid conversion isolates also behave very differently (Ryall et al., 2014). The mucoid phenotype showed enhanced microcolony formation and tobramycin resistance compared to the non-mucoid form of *P. aeruginosa* (Hentzer et al., 2001). We now show that all of the strains caused varying degrees of antagonism, virulence, and inflammation and demonstrate the strain-specific interactions between organisms in microbial communities. It is vital for studies of this nature to include a variety of clinical strains with reference strains in order to get a thorough picture of the nature of species interaction.

The well-diffusion assay, although relatively basic, provided a good starting point for testing relationships between microorganisms and showed that all the *P. aeruginosa* strains tested inhibited the *A. fumigatus* strains, with zones of clearance apparent around all wells. Antagonism is defined as the action of any organism that suppresses or interferes with the normal growth and activity of another organism. Antagonism was evident in biofilm growth patterns where different strain combinations caused varying degrees of disruption to overall biofilm biomass generation. The crystal violet biofilm stain is widely published as a measure of total biomass (O’Toole and Kolter, 1998); however, when studying polymicrobial biofilms, tracking growth of each species individually is essential. We performed species-specific qPCR to distinguish between *A. fumigatus* and *P. aeruginosa* in co-cultured biofilms and found that both *A. fumigatus* and *P. aeruginosa* were negatively impacted by the interaction revealing a mutually antagonistic relationship. We have confirmed this across a range of reference strains and clinical isolates. It is worth highlighting that this PMA qPCR method is a very useful method for studying polymicrobial biofilms. The inclusion of a PMA-treatment step is important for accurate qPCR based enumeration, removing non-viable *A. fumigatus* and *P. aeruginosa* from biofilms and resulting in a more accurate count of viable pathogens. The qPCR-PMA+ assays offer a valuable diagnostic tool and could prove useful in monitoring the pathogen bioburden during/post antibiotic treatment. Overall, these reductionist approaches would be enhanced via microscopic analysis of co-cultured biofilms of these two organisms in a flow cell system (Murphy and Caraher, 2015).

We observed that *A. fumigatus* CSNs inhibited *P. aeruginosa* biofilm formation and this has not been shown previously. *A. fumigatus* hyphae produce a number of toxins including helvolic acid, fumagillin and gliotoxin (Amitani et al., 1995; Hogan et al., 1996). Increased concentrations of these toxins in 48 h and 72 h CSN may account for the increased inhibitory effect on *P. aeruginosa* biofilms. Manavathu and Vazquez (2014) examined the antibacterial activity of *A. fumigatus* CSN on *P. aeruginosa* planktonic growth and showed that at 48 h *A. fumigatus* CSN contained a heat stable compound which inhibited *P. aeruginosa* growth in a bacteriostatic fashion (Manavathu and Vazquez, 2014). We demonstrate that *A. fumigatus* produces an anti-pseudomonal and anti-biofilm agent at 48 h and 72 h of growth. Gt is a redox-active metabolite that is produced by *A. fumigatus* at 48 h and 72 h (Reeves et al., 2004), and its biosynthesis is directed by the *gli* gene cluster. Employing a *gli* gene mutant strain of *A. fumigatus*, we showed that CSNs from the mutant strain did not possess any inhibitory qualities against *P. aeruginosa* and exposure to CSNs from the complement strain restored inhibition suggesting gliotoxin is the main inhibitory agent secreted by *A. fumigatus*.

Gt belongs to the epipolythiodioxopiperazine class of secondary metabolites produced by *A. fumigatus* and it is characterized by a disulfide bridge known to be essential for activity (Chai and Waring, 2000). Gt displays immunosuppressive properties (Sutton et al., 1994; Schlam et al., 2016) and was previously considered for treatment of bone marrow transplant patients (Müllbacher et al., 1987; Mullbacher et al., 1988). Gt can also inhibit macrophage function (Bertout et al., 2002), induce apoptotic cell death in macrophages (Waring, 1990), block T and B cell activation and generate cytotoxic cells (Müllbacher and Eichner, 1984; Eichner et al., 1986; Sutton et al., 1996). Gt and its analogs possess antimicrobial activity against a number of microorganisms including methicillin-resistant *S. aureus*, extended-spectrum beta-lactamase-producing *E. coli*, vancomycin-resistant *Enterococcus faecalis* and *C. albicans* (Li et al., 2006; Svahn et al., 2012). While Gt would be too toxic for use as an anti-pseudomonal, Gt derivatives that maintain anti-biofilm activity without host cell toxicity should be evaluated. Although Gt is unlikely as a potential therapeutic, its antimicrobial effects against multiple species demonstrated here suggests that *A. fumigatus* colonization and Gt production in the airways may cause disruption of the airway microbiome and this is yet to be fully explored.

Co-infections with these mutual antagonists caused greater *G. mellonella* mortality than single *P. aeruginosa* infections. *G. mellonella* possess an innate immune system homologous to that of mammals (Bergin et al., 2005). Pre-exposure of larvae to non-lethal doses of *A. fumigatus* conidia increased the mortality of *G. mellonella* when co-infected with *P. aeruginosa* above that caused by *P. aeruginosa* alone. *A. fumigatus* produces many immunoregulatory toxins that could suppress the immune system of *G. mellonella*, making the larvae more susceptible to *P. aeruginosa* infection. Gt inhibits *G. mellonella* hemocyte phagocytosis and ROS formation (Renwick et al., 2007). Alternatively, the interaction between *A. fumigatus* and *P. aeruginosa* could cause a more virulent infection. Interestingly the isolates, which had originally co-colonized in the same patient showed no increase in larvae mortality when co-infecting, suggesting that these isolates may have adapted to co-exist in the same environment. Overall, these findings reveal bacterial–fungal interactions may contribute to increased pathogenicity in a strain-specific manner.

*Pseudomonas aeruginosa* is widely accepted to stimulate inflammation via MAP kinase activation (Beaudoin et al., 2013). *A. fumigatus* has been reported to have both pro-inflammatory and immunosuppressive effects (Margalit and Kavanagh, 2015; King et al., 2016; Zhang et al., 2017). The *A. fumigatus* strains examined in this study stimulated the secretion of pro-inflammatory cytokines from CFBE cells. While it might be expected that the combination of both pro-inflammatory pathogens would have an additive effect on inflammation, we have shown no additive inflammation when cells were exposed to both *A. fumigatus* and *P. aeruginosa* for the majority of combinations tested. We acknowledge that these polymicrobial effects on host immune dysregulation are likely to be quite complex, thus we attempted to explore possible pathways for the lack of additional inflammatory cytokine secretion. Mutual antagonism between the pathogens may reduce the bioburden that CF lung cells were exposed to potentially providing a benefit to the co-colonizers and/or it may be an adaptation of the co-colonizers allowing them to co-colonize and evade the immune system of the host and survive in the airways. Another likely scenario is that both pathogens saturate the ERK and p38 MAP kinase pathway for activating cytokine production. MAPKs facilitate signaling associated with a number of cellular activities such as cell survival, cell death, proliferation, differentiation, and inflammation (Cowan and Storey, 2003). In this study, we found that *P. aeruginosa* and *A. fumigatus* both signal cytokine production in CF epithelial cells via activation of the ERK and p38 MAP kinase pathways. Therefore the lack of synergistic inflammatory response observed due to co-infection may be due to saturation of signaling pathways. The complexity of these multifactorial interactions, will require much more examination to elucidate these pathways and identify other potential mechanisms by which these pathogens are able to evade the host immune system, establish favorable conditions and chronically persist in the CF airways.

Overall, we show for the first time that *P. aeruginosa* and *A. fumigatus* have mutually antagonistic effects at the formation stage of biofilm development in the mixed species biofilm. Other studies have noted the anti-*fumigatus* effects of *P. aeruginosa* in planktonic (Briard et al., 2016) and biofilm (Mowat et al., 2010; Briard et al., 2015; Ferreira et al., 2015; Shirazi et al., 2016) forms of growth, however, it has not been previously shown that *P. aeruginosa* also suffers from this cross-kingdom interaction. Gt secreted by *A. fumigatus* is anti-pseudomonal and anti-biofilm and could be investigated with a view to finding safer analogs as therapeutics against antibiotic resistant biofilms in CF airway infections and many other infections. Bacterial–fungal interactions may also contribute to increased pathogenicity, which could have a significant impact on the treatment of these pathogens in patients with CF. These co-infecting microbes contribute to an altered inflammatory response that could aid evasion of the immune system and chronic colonization resulting in detrimental effects on a CF patient’s health status.

## Author Contributions

ER was involved in study design, data analysis, data interpretation, and writing and editing the manuscript. SM was involved in study design, data interpretation, and writing and editing the manuscript. JR was involved in study design, data interpretation, and editing the manuscript. SD was involved in study design, provision of gliotoxin mutant and editing the manuscript. PG was involved in study design, data interpretation, and manuscript editing.

## Conflict of Interest Statement

PG has served on advisory boards and received honoraria from Vertex, Novartis, Pharmaxis, and PTC pharmaceuticals in the past however none of these organizations have contributed financially or in any other way to this work. The other authors declare that the research was conducted in the absence of any commercial or financial relationships that could be construed as a potential conflict of interest. The reviewer AP and handling Editor declared their shared affiliation.
