# Supplementary material for: Aspergillus fumigatus Inhibits Pseudomonas aeruginosa in Co-culture: Implications of a Mutually Antagonistic Relationship on Virulence and Inflammation in the CF Airway
Source: Front Microbiol. 2018 Jun 5;9:1205. doi: 10.3389/fmicb.2018.01205 (PMC5996130; doi:10.3389/fmicb.2018.01205)
Supplement: Supplementary file 1 [file Data_Sheet_1.docx]

**Supplementary Table 1:** Primers and probes used in this study for the detection and quantification of *P. aeruginosa* and *A. fumigatus*

| Primer name | Sequence 5’-3’ | Reference |
| --- | --- | --- |
| gyrB-Forward | CCTGACCATCCGTCGCCACAAC | (Qin et al., 2003) |
| gyrB-Reverse | CGCAGCAGGATGCCGACGCC | (Qin et al., 2003) |
| gyrB-Probe | FAM-CCGTGGTGGTAGACCTGTTCCCAGACC-BHQ | (Anuj et al., 2009) |
| ecfX-Forward | CGCATGCCTATCAGGCGTT | (Anuj et al., 2009) |
| ecfX-Reverse | GAACTGCCCAGGTGCTTGC | (Anuj et al., 2009) |
| ecfX-Probe | YAK-ATGGCGAGTTGCTGCGCTTCCT-BHQ | (Anuj et al., 2009) |
|  |  |  |
| ITS-Forward | GCCCGCCGTTTCGAC | (Walsh et al., 2011) |
| ITS-Reverse | CCGTTGTTGAAAGTTTTAACTGATTAC | (Walsh et al., 2011) |
| ITS-Probe | FAM-CCCGCCGAAGACCCCAACATG-TAM | (Walsh et al., 2011) |
|  |  |  |
| ctrA- Forward | GCTGCGGTAGGTGGTTCAA | (Corless, Guiver, Borrow, Fox, & Kaczmarski, 2001) |
| ctrA- Reverse | TTGTCGCGGATTTGCAACTA | (Corless et al., 2001) |
| ctrA-Probe | FAM-CATTGCCACGTGTCAGCTGCACAT-TAM | (Corless et al., 2001) |

Supplementary Figure 1

**Supplementary Figure 1: Zones of inhibition in well-diffusion assay**

*P. aeruginosa* mucoid isolate PA27 (A & B), non-mucoid isolate PA27N (C & D), co-colonisers PAco1 and PAco2 (E & F) in the wells of the MEA plate and the *A. fumigatus* isolates AF1 (A & C) and AF2 (B & D), co-colonisers AFco1 and AFco2 (E & F) spread on the lawn of the plate. Best images of three replicate assays performed are shown.

Supplementary Figure 2

**Supplementary Figure 2: Percentage survival of *G. mellonella* when infected with *A. fumigatus* conidia**

The percentage survival of *G. mellonella* when infected with ten-fold serial dilutions of *A. fumigatus* (1 – 1x 10^7^ conidia/larva) A) AFref2 reference strain B) AFco1 C) AF1 D) AF2. Experiments were carried out on three independent occasions and error bars represent standard deviation.

Supplementary Figure 3

#### Supplementary Figure 3: The cytotoxic effect of P. aeruginosa and A. fumigatus infection in CFBES cells

Percentage of viable CFBE cells after infection with A) *P. aeruginosa* PAref MOI of 30:1, 50:1, 80:1 and 100:1 and B) *A. fumigatus* AFref1 MOI 1:1, 2:1, 5:1, 10:1 compared to uninfected CFBE cells (control) measured by MTT assay. Experiments were carried out on three occasions in duplicate and error bars represent standard deviation. **** p ≤ 0.0001.

Supplementary Figure 4

**Supplementary Figure 4: Effect of co-infection with *P. aeruginosa* cells and *A. fumigatus* conidia on IL-6 and IL-8 secretion in the presence of MAPK inhibitors**

Percentage reduction of IL-6 pg/ml secretion from CFBE cells in response to exposure of co-culture of *P. aeruginosa* and *A. fumigatus* A) PA27 + AF1 B) PA27 + AF2 C) PA27N + AF1 D) PA27N + AF2 and and IL-8 pg/ml secretion E) PA27 + AF1 F) PA27 + AF2 G) PA27N + AF1 H) PA27N + AF2 in the presence of MAPK inhibitors. Experiments were carried out on three independent occasions in triplicate and error bars represent standard deviation. * p ≤ 0.05, *** p ≤ 0.001, **** p ≤ 0.0001
